# A Case Report of Allergic Contact Dermatitis due to *Mandragora* Radix

**DOI:** 10.1155/2015/591438

**Published:** 2015-08-04

**Authors:** Sevim Baysak, Müzeyyen Gönül, Damla Atacan, Can Ergin

**Affiliations:** Department of Dermatology, Yıldırım Beyazıt Training and Research Hospital, 06120 Ankara, Turkey

## Abstract

An 82-year-old male presented with rash, burning, and itching on his knees that had started 4 days after the local application of *Mandragora Radix* sap for 3 consecutive days. A dermatological examination revealed erythematous, edematous, and scaly plaque lesions on the patient's knees. An open application test with *M. Radix* was performed, and the patient was diagnosed with allergic contact dermatitis due to *M. Radix*. *Mandragora* species, which belong to the Solanaceae family, have sedative, aphrodisiac, emetic, analgesic, and anesthetic properties. To the best of our knowledge, only one case of *M. Radix*-induced allergic contact dermatitis has been previously reported.

## 1. Introduction

Herbs have been used for medical purposes since ancient times [1]. The use of herbs and alternative therapies for mild and self-limiting diseases is becoming increasingly popular worldwide [2]. One study reported that the rate of complementary and alternative medicine use among patients with dermatologic disease in Turkey was 33.5% [3].* Mandragora* species, which belong to the Solanaceae family, have sedative, aphrodisiac, emetic, analgesic, and anesthetic properties [4].* Mandragora* plants, which are native to Central Asia, North India, Southern Europe, and the Mediterranean Basin, contain scopolamine, hyoscyamine, and atropine.

## 2. Case

**Figure 1:**
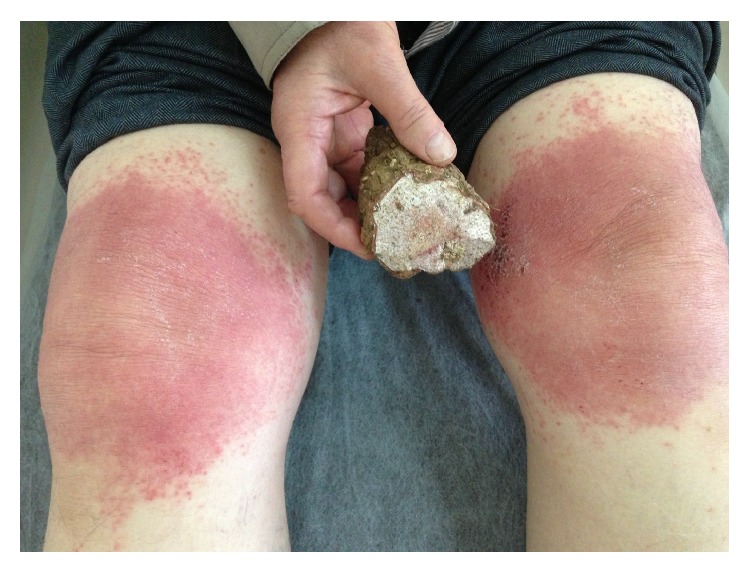
Erythematous, edematous, and mild scaly plaques exceeding the application border on the skin and Mandragora Radix.

**Figure 2:**
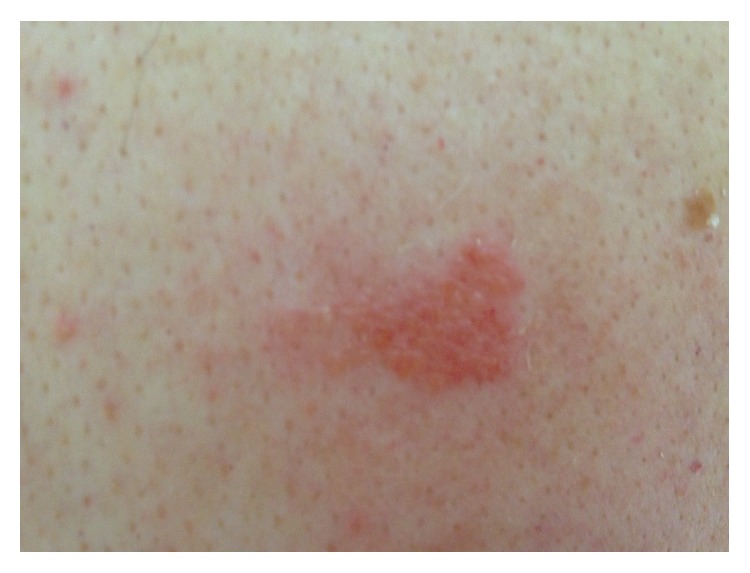
Positive open patch test result with Mandragora Radix.

An 82-year-old male presented with rash, burning, and itching on both knees. The patient had applied* M. Radix* sap to his knees for arthralgia for 3 consecutive days, and the lesions appeared on the fourth day. He denied any prior contact with* M. Radix*. The patient had gonarthrosis but was otherwise healthy. Dermatological examination findings were normal except for erythematous, edematous, and scaly plaque lesions exceeding the borders of the area on which the* M. Radix* sap had been applied (Figure 1). An open application test (OAT) was performed because of the potential for a severe allergic or irritant reaction during the closed patch test. A small amount of* M. Radix* sap was directly applied to a 1 cm^2^ area of skin on the patient's upper arm. No reaction was observed for 30 minutes. The application was not repeated because the primary allergic reaction was severe. The OAT result was positive at 48 hours. No change in reaction severity was observed at 72 hours. The patient was diagnosed with allergic phytocontact dermatitis based on the clinical findings and the OAT result (Figure 2). The lesions resolved with topical corticosteroid therapy. To the best of our knowledge, only one case of* M. Radix*-associated allergic contact dermatitis has been previously reported [5].

## 3. Discussion

Herb-related adverse effects may cause a wide range of health problems, including life-threatening reactions, multisystemic diseases, and increased hospitalization rates [6]. Many cutaneous adverse effects secondary to herbs have been reported, including allergic and irritant contact dermatitis, Stevens-Johnson syndrome, anaphylaxis, photosensitivity, and pellagra [7, 8]. Irritant phytocontact dermatitis, allergic phytocontact dermatitis, or phytophotocontact dermatitis may occur after the topical application of certain plants [9].

There are various species of* Mandragora*; however,* Mandragora officinarum* might be responsible for most reactions because it is the most common species in the Mediterranean Basin [5]. We believe that the reaction in our patient was allergic contact dermatitis for three reasons. First, the patient had no subjective complaints for 30 minutes after the OAT. Second, a positive reaction was detected at 48 hours. Third, the reaction severity did not decrease at 72 hours. To the best of our knowledge, this is the second reported case of allergic contact dermatitis due to* M. Radix*. The substance responsible for the reaction in our patient is unknown because more than 80 different substances have been isolated from various species of* Mandragora* [4].

There are two phases in the development of allergic contact dermatitis: the afferent phase, in which first contact with the substance and sensitization occur, and the efferent phase, in which a T cell-mediated delayed hypersensitivity reaction occurs upon second contact with the substance. The afferent phase lasts for 10 to 15 days in most cases and is asymptomatic. This phase may be short, and allergic contact dermatitis can be induced even after a single skin contact with a strong hapten in unsensitized individuals. In such cases, the two phases occur in a single step [10, 11]. The reaction in our patient might have occurred in a single step because he reported no previous contact with* M. Radix*. Other possible mechanisms in our case include sensitization and induction of the afferent phase by previous contact with an allergen in a* Mandragora* plant or the development of a delayed reaction by cross-reaction after sensitization caused by previous contact with a Solanaceae family member such as potato or tomato [5].

Our case illustrates the potential side effects of herbal therapies. Obtaining a detailed medical history, including potential skin reactions to herbal products, would enable clinicians to treat patients successfully. Those who use alternative medicine must consider the possibility of allergic contact dermatitis due to* M. Radix*.

## References

[1] Chidiac E. J., Kaddoum R. N., Fuleihan S. F. (2012). Mandragora: anesthetic of the ancients. *Anesthesia and Analgesia*.

[2] Wilczek A., Sticherling M. (2006). Concomitant psoriasis and bullous pemphigoid: coincidence or pathogenic relationship?. *International Journal of Dermatology*.

[3] Gönül M., Gül Ü., Çakmak S. K., Kılıç S. (2009). Unconventional medicine in dermatology outpatients in Turkey. *International Journal of Dermatology*.

[4] Tsiligianni I. G., Vasilopoulos T. K., Papadokostakis P. K., Arseni G. K., Eleni A., Lionis C. D. (2009). A two cases clinical report of mandragora poisoning in primary care in Crete, Greece: two case report. *Cases Journal*.

[5] Gönül M., Çakmak S. K. (2013). A case of allergic skin reaction to mandragora radix. *Journal of Clinical & Experimental Dermatology Research (Special Issue)*.

[6] Bedi M. K., Shenefelt P. D. (2002). Herbal therapy in dermatology. *Archives of Dermatology*.

[7] Schempp C. M., Müller K. A., Winghofer B., Schöpf E., Simon J. C. (2002). St. John's wort (*Hypericum perforatum* L). A plant with relevance for dermatology. *Der Hautarzt*.

[8] Ernst E. (2000). Adverse effects of herbal drugs in dermatology. *British Journal of Dermatology*.

[9] Goon A. T. J., Goh C. L. (2011). Plant dermatitis: Asian perspective. *Indian Journal of Dermatology*.

[10] Vocanson M., Hennino A., Rozières A., Poyet G., Nicolas J.-F. (2009). Effector and regulatory mechanisms in allergic contact dermatitis. *Allergy*.

[11] Sastre J. (2010). Molecular diagnosis in allergy. *Clinical and Experimental Allergy*.

